# Whisker Deprivation Drives Two Phases of Inhibitory Synapse Weakening in Layer 4 of Rat Somatosensory Cortex

**DOI:** 10.1371/journal.pone.0148227

**Published:** 2016-02-03

**Authors:** Melanie A. Gainey, Renna Wolfe, Olivia Pourzia, Daniel E. Feldman

**Affiliations:** Department of Molecular and Cellular Biology and Helen Wills Neuroscience Institute, University of California, Berkeley, California, United States of America; Radboud University Nijmegen, NETHERLANDS

## Abstract

Inhibitory synapse development in sensory neocortex is experience-dependent, with sustained sensory deprivation yielding fewer and weaker inhibitory synapses. Whether this represents arrest of synapse maturation, or a more complex set of processes, is unclear. To test this, we measured the dynamics of inhibitory synapse development in layer 4 of rat somatosensory cortex (S1) during continuous whisker deprivation from postnatal day 7, and in age-matched controls. In deprived columns, spontaneous miniature inhibitory postsynaptic currents (mIPSCs) and evoked IPSCs developed normally until P15, when IPSC amplitude transiently decreased, recovering by P16 despite ongoing deprivation. IPSCs remained normal until P22, when a second, sustained phase of weakening began. Delaying deprivation onset by 5 days prevented the P15 weakening. Both early and late phase weakening involved measurable reduction in IPSC amplitude relative to prior time points. Thus, deprivation appears to drive two distinct phases of active IPSC weakening, rather than simple arrest of synapse maturation.

## Introduction

Inhibitory synaptogenesis occurs over a prolonged postnatal period in cerebral cortex. In primary sensory cortex, this period extends from birth to at least 5 weeks of age [1–6], during which the physiological strength of inhibition gradually increases [7–10]. Inhibitory synaptogenesis and maturation is thought to be strongly driven by sensory experience, because sensory-deprived animals often show weaker inhibition and persistence of immature synaptic phenotypes [11–16]. Precisely how deprivation affects inhibitory synapse maturation is unclear. Understanding this process may be relevant for understanding the development of abnormal inhibitory transmission in disorders such as epilepsy [17], autism [18] or schizophrenia [19].

We investigated how sensory deprivation regulates inhibitory synapse development in layer (L) 4 of rat whisker somatosensory cortex (S1). Whisker deprivation is known to reduce L4 inhibitory synapse number, interneuron excitability and spiking, and functional inhibition within whisker receptive fields [5,11–13,15–16,20]. In prior studies of inhibitory synapse physiology, long-duration whisker deprivation from postnatal day (P) 7 to P28 was shown to cause a reduction in spontaneous and evoked inhibitory synaptic transmission onto L4 excitatory cells, measured at P28 [13,16]. The dynamics of inhibitory weakening, however, remain unclear. The dominant model is that whisker deprivation arrests the normal development of inhibitory synapses, so that deprivation effects slowly accumulate, relative to age-matched controls, with increasing duration of deprivation. Alternatively, deprivation could drive one or more active weakening processes, which could be confined to specific, distinct periods of development. Brief whisker deprivation from P5 to P9-11 does not affect L4 inhibitory synapse physiology [21], suggesting that experience may primarily affect later synapse development, after onset of active whisking at P12 [22].

To address this issue, we measured the dynamics by which deprivation affects L4 inhibitory synapse physiology in rat S1. We plucked the D row of whiskers beginning at P7, which is the same manipulation that is known to weaken inhibitory transmission measured at P28 [13]. We measured the effects of this deprivation on L4 inhibitory synaptic transmission at multiple time points up to P30, in deprived (D-row) and spared (B-row) barrels in acute S1 slices. We assessed synaptic transmission by measuring spontaneous miniature and locally evoked inhibitory postsynaptic currents (mIPSCs and eIPSCs) in L4 excitatory neurons. Despite continuous whisker deprivation from P7, IPSCs developed normally until P15, when IPSC amplitude was transiently reduced for 1 day. Remarkably, IPSCs recovered at P16 despite ongoing deprivation, and remained normal until a second phase of weakening began at P22. This later phase was sustained until at least P30. When deprivation onset was delayed until P12, the early P15 weakening failed to occur. Early and late phases of IPSC weakening differed in column specificity and effect on paired pulse ratio. These findings suggest that deprivation drives two distinct phases of IPSC weakening, rather than a simple arrest of synapse maturation.

## Materials and Methods

Experimental procedures were approved by the UC Berkeley Institutional Animal Care and Use Committee. Long-Evans rats (aged P7-30) were housed in litters in standard laboratory cages until P21, when they were weaned and housed in groups of 2–3 littermates. A subset of rats had whiskers D1-D6 and gamma removed from the right side of the snout by plucking under transient isoflurane anesthesia (3%, in 2L/min oxygen). Whisker plucking is a robust, innocuous manipulation that reduces sensory input without damaging peripheral afferents [23]. Plucking began at either age P7 or P12, and continued on alternating days until the day of recording. A total of 105 rats were used (17 with normal whisker experience and 88 with D-row whisker deprivation).

### Slice preparation

Rats were anesthetized with isoflurane and decapitated, and the brain was quickly removed and placed in ice cold oxygenated Ringer’s solution (containing in mM: NaCl 119, NaHCO_3_ 26.2, D-(+)-glucose 11, MgSO_4_ 1.3, KCl 2.5, NaH_2_PO_4_ 1.0, CaCl_2_ 2.5, pH 7.2, bubbled with 5% CO_2_/95% O_2_). Slices (350 μm thickness) of the left cortical hemisphere were cut in the “across-row” plane of section, oriented 45^0^ towards coronal from the sagittal plane. In this plane of section, slices containing the posteromedial barrel subfield (PMBSF) include one barrel column from each of five whisker barrel rows, A through E. This enables A-E columns to be identified in living slices and used to target physiological recordings [24,25]. After cutting, slices were incubated at 32° C for 30 min, and then maintained at room temperature until recording (1–7 hrs later).

### Electrophysiological recording

Whisker barrels in cortical layer 4 were visualized by transillumination to identify whisker-related columns and cortical layers. Using infrared differential interference contrast (IR-DIC) at 40× magnification, pyramidal-shaped neurons in L4 were selected for whole-cell voltage-clamp recording. Recordings were made using a Multiclamp 700B amplifier (Molecular Devices), low-pass filtered at 2 kHz, and sampled at 5 kHz using a 12-bit data acquisition board (National Instruments, Austin, TX). Electrophysiological data were collected using custom acquisition routines in Igor Pro (Wavemetrics, Lake Oswego, OR). Recordings were performed at 30–31° C in oxygenated Ringer’s solution (~ 2 mL/min). Pipette resistance was 2–4 MΩ. Series resistance was 10–20 MΩ. Cells were discarded if series or input resistance changed more than 15% during recording, or if the holding current to maintain V_hold_ = -70 mV exceeded -200 pA.

Inhibitory postsynaptic currents (IPSCs) were measured in voltage clamp at 0 mV, using Cs gluconate internal solution (in mM: D-gluconic acid 108, Cesium OH 108, HEPES 20, TEACl 5, NaCl 2.8, EGTA 0.4, Na-GTP 0.3, Mg-ATP 4.0, Na_2_-phosphocreatine 10.0; adjusted to pH 7.2 with CsOH). Predicted chloride reversal potential using these solutions was -71 mV. For all experiments, the bath contained the glutamate receptor antagonists D-AP5 (50 μM) and NBQX (10 μM). All drugs were from Tocris Biosciences (Ellisville, MO).

### Spontaneous miniature IPSCs (mIPSCs)

**Fig 1:**
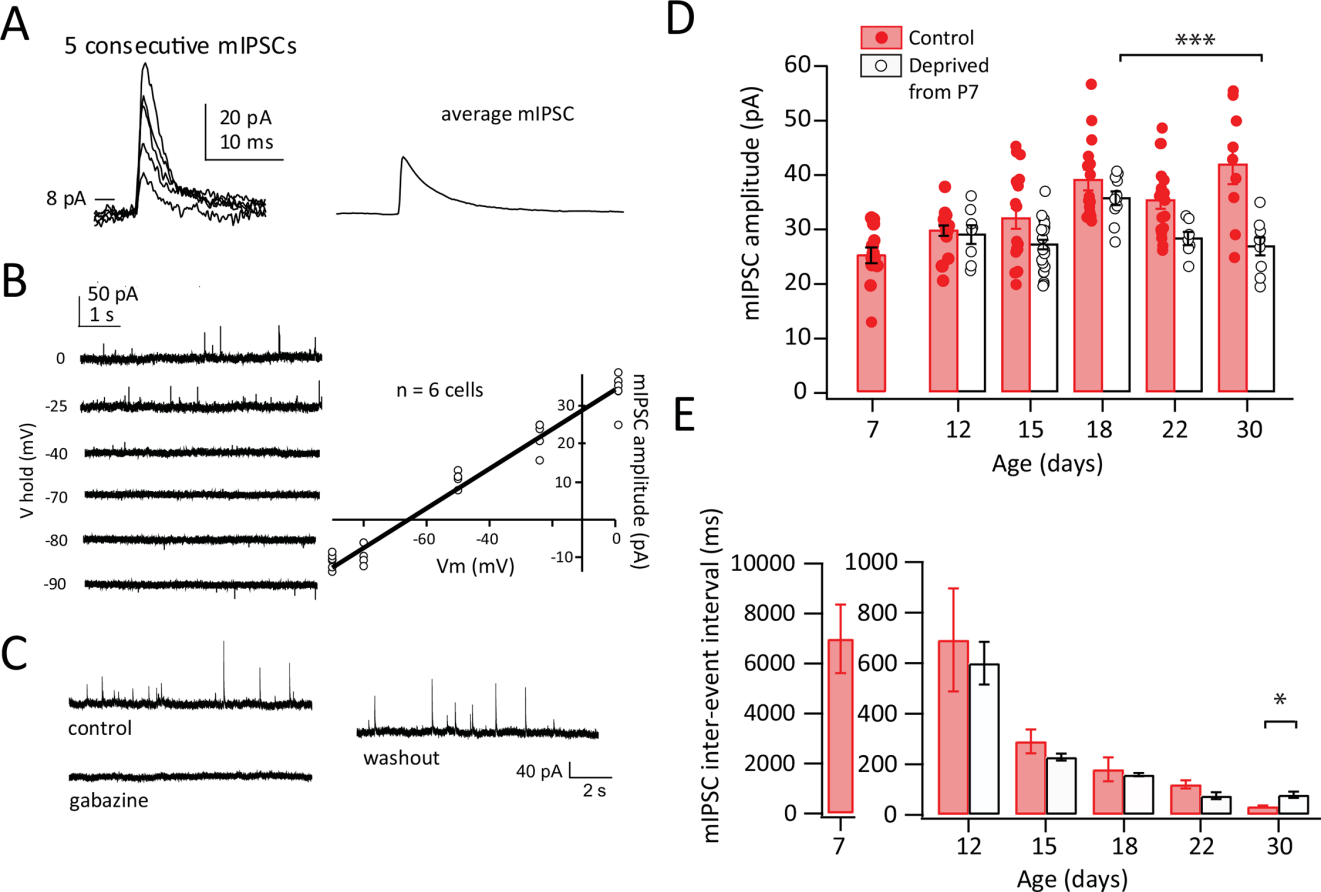
Development of mIPSCs and effect of D-row whisker deprivation from P7. **A**, Individual mIPSCs and mean mIPSC from a L4 neuron, recorded at Vhold = 0 mV. Detection threshold was 8 pA. **B**, mIPSCs recorded at various holding potentials. Right, I-V plot for 6 cells showing reversal potential. **C**, Example cell showing reversible blockade of mIPSCs by gabazine (1.5 μM). **D**, Mean mIPSC amplitude at different ages, for cells in D columns of control rats (filled) and deprived D columns of D-row whisker deprived rats (open). Each circle is a neuron. Bars show mean ± SEM. **E**, Mean mIPSC inter-event interval. Conventions as in (D). * = p < 0.05, ** = p < 0.01, *** = p < 0.005, **** = p < 0.001.`

Spontaneous miniature IPSCs (mIPSCs) were recorded as outward currents at 0 mV in glutamate receptor antagonists plus tetrodotoxin (TTX) (500 nM). Standard peak-to-peak recording noise was ~ 14 pA [range 10–18 pA]. However, mIPSCs as small as 8 pA could be reliably detected because of their characteristic time course. mIPSC event detection was performed semi-automatically using a template matching method in Axograph X (AxoGraph Scientific, Sydney Australia). Approximately 500 mIPSCs were analyzed per cell. All mIPSC analysis was performed blind to the animal’s sensory experience. mIPSC reversal potential (Fig 1B) was determined by linear regression on mean mIPSC amplitude at 0, -25, -50, -80, and -90 mV holding potentials.

### Minimal evoked IPSCs

A bipolar extracellular stimulating electrode (~100 μm tip spacing) (FHC, Bowdoin ME) was placed at the bottom of a L4 barrel, at a depth of 200 μm below the cut surface of the slice. Evoked IPSCs were recorded from neurons in the same barrel at 0 mV holding potential, in response to constant-current stimulation pulses (200 μs; 25 ms interstimulus interval). AMPA, NMDA, and GABA_B_ receptors were blocked with D-AP5 (50 μM) and NBQX (10 μM) and saclofen (100 μM) in the Ringer’s solution.

For each cell, a threshold stimulus intensity was identified that yielded the smallest IPSC above background recording noise. The stimulation intensity was then adjusted to just above response threshold, to evoke IPSCs at a failure rate of ~ 50%. Because minimal IPSCs were large (~15–60 pA) relative to the detection threshold, it was usually straightforward to recognize successes from failures. Minimal IPSCs were characterized from 50–100 sweeps recorded at this stimulation intensity. In off-line analysis, we calculated the peak amplitude of the IPSC in each sweep (amplitude in a 5-ms window centered on average IPSC peak minus current amplitude in an equal-duration, prestimulus window). Successes and failures were distinguished by comparing the distributions of IPSC amplitude measured during stimulation sweeps and non-stimulation sweeps. The distribution of negative amplitudes measured in non-stimulation sweeps was mirror-replicated around 0 pA, and the resulting “noise distribution” was fit with a Gaussian. IPSC potency for each cell was defined as the mean of all IPSCs during stimulation sweeps that were greater than two times the standard deviation of the Gaussian noise distribution (i.e., as the mean amplitude of successes). Only recordings with 40–60% failure rates by off-line analysis were included in the data set.

### Histological verification of L4 neuron type

The study design assumed that recorded neurons are excitatory. To test this assumption, L4 neurons were filled with biocytin (0.25% w/v in internal) during whole-cell recording in a subset of experiments. After recording, slices were fixed in 4% paraformaldehyde in 0.1 M phosphate buffer (pH 7.4). Slices were re-sectioned (100 μm thickness) on a freezing microtome, then reacted with streptavidin-fluorescein 0.2% (Vector Labs, Burlingame, CA) in 0.1 M PB for 3 days at 4°C. After mounting and coverslipping (Vectashield), dendritic morphology was examined under 40 x magnification to determine cell type.

### Statistics

All t-tests and reported p-values are Bonferroni-corrected, unless otherwise noted.

## Results

We used whole-cell voltage clamp recording to measure inhibitory synaptic currents in L4 of S1 acute brain slices from P7-30 Long-Evans rats. Slices were cut in the oblique, ‘across row’ plane of section, and transillumination was used to identify L4 barrels corresponding to the A-E whisker rows [24,25]. Neurons were patched in the D or B barrel in L4, using cesium gluconate internal to achieve adequate voltage clamp. Our goal was to record from excitatory L4 neurons. Because the presence of cesium prevented us from identifying excitatory neurons by spiking pattern, we targeted recordings to neurons with pyramidal-shaped somata, and determined targeting accuracy by filling a subset of 58 neurons with biocytin during recording. Visualization of neuronal morphology in streptavidin-reacted slices showed that 54/58 neurons (93%) had dendritic spines characteristic of excitatory cells. 58% of recovered neurons were spiny stellate cells and 42% were pyramidal cells, which are the two major excitatory cell classes in L4 of S1 [26]. Recordings in all experiments were targeted to presumed excitatory cells using this method.

### Development of mIPSCs in L4

We first assayed development of L4 inhibitory synapse function using spontaneous miniature inhibitory postsynaptic currents (mIPSCs). mIPSCs were recorded from L4 neurons in voltage clamp in TTX (500 nM) to block action potentials and NBQX (10 μM) and APV (50 μM) to block fast excitatory synaptic transmission. mIPSCs appeared as spontaneous outward currents at 0 mV (Fig 1A). mIPSCs reversed at -66 ± 0.3 mV (n = 6 cells), close to the calculated chloride reversal potential of -71 mV for these solutions (Fig 1B). mIPSCs were completely and reversibly blocked by the GABA-A antagonist gabazine (1.5 μM) (n = 5 cells, Fig 1C).

To characterize normal development of inhibitory synapses, we measured mIPSCs at different ages in L4 barrels in whisker-intact rats (Fig 1D and 1E). mIPSCs were identified with a template matching algorithm using a 8.0 pA amplitude threshold. Mean mIPSC amplitude and interevent interval (IEI) were calculated for each cell from ~ 500 mIPSCs. 9–18 cells (from 2–3 rats from separate litters) were analyzed at each age. Mean mIPSC amplitude increased significantly between P7 and P30 (ANOVA, p < 0.001), with a significant increase between P7 and P18 (Fisher’s PLSD; p<0.001) and more stable amplitude between P18 and P30 (Fig 1D). mIPSC inter-event interval [IEI] decreased significantly from P7 to P30 (ANOVA, p< 0.001), reflecting an increase in mean frequency (Fig 1E). These results are consistent with prior findings of increased GABAergic synapse number and efficacy during postnatal weeks 1–3 in S1 [5,6,10,13] and visual cortex (V1) [9].

### Effect of whisker deprivation from P7 on mIPSC development

To determine how whisker deprivation affects development of L4 mIPSCs, we deprived the D-row whiskers by plucking beginning at P7. Deprivation was maintained continuously by re-plucking every 2–3 days until the date of recording (Fig 1D). Whisker plucking reduces sensory-evoked activity in the corresponding S1 column [27] and drives robust plasticity of whisker-evoked spiking responses and synaptic properties in L2/3 of deprived columns [28–30]. It has previously been shown that continuous whisker deprivation from P7 in GAD67-GFP mice causes weakened L4 inhibitory synapse efficacy measured at P28 [13]. Here, we examined the dynamics of this weakening, by comparing mIPSCs at different ages in D columns of D-row deprived rats (n = 8–24 cells, 27 rats per age) (Fig 1D and 1E).

Whisker deprivation did not simply arrest development of mIPSC amplitude, but instead altered mIPSC amplitude in a complex pattern across age. At P12, mIPSC amplitude in deprived columns was indistinguishable from control, whisker-intact rats. mIPSC amplitude increased significantly in deprived columns between P12 and P18 (Fisher’s PLSD, p<0.005), similar to controls. Within this period, there was a noticeable but non-significant trend toward reduced mIPSC amplitude at P15 (control: 31.7 ± 2.0 pA, n = 18 cells; deprived: 26.9 ± 0.9 pA, n = 26 cells). While no effect of deprivation was observed at P18, beginning at P22 a significant reduction in mIPSC amplitude was observed in deprived columns relative to controls (2-way ANOVA, p < 0.001). This was observed at both P22 (control: 35.1 ± 1.6 pA, n = 18 cells; deprived: 28.0 ± 1.2 pA, n = 7 cells) and P30 (control: 41.6± 3.7pA, n = 9 cells; deprived: 26.6 ± 1.6 pA, n = 9 cells; p<0.015 for both ages, Bonferroni-corrected t-test). Within deprived columns, mIPSC amplitude reduced significantly between P18 and P30 (Bonferroni-corrected t-test, p < 0.005). This pattern suggests that the reduction in mIPSC amplitude observed previously at P30 [13] does not reflect a simple arrest of mIPSC development. Instead, mIPSC amplitude develops roughly normally through P18, and a dynamic reduction in mIPSC amplitude only begins at P22, which is 15 days after the onset of deprivation.

In the same recordings, mIPSC IEI was not significantly different in deprived rats compared to control rats, except for a significant increase at P30 (control: 33.1 ± 2.3 ms, n = 9 cells; deprived: 79.6 ± 12.4 ms, n = 10 cells; p<0.02, Bonferroni-corrected t-test). (Fig 1E). Within deprived rats, IEI decreased with age from P12 to P30 (ANOVA, p<0.001), similar to the pattern observed in controls.

### Whisker deprivation drives two distinct phases of mIPSC weakening at P15 and at P22-30

**Fig 2:**
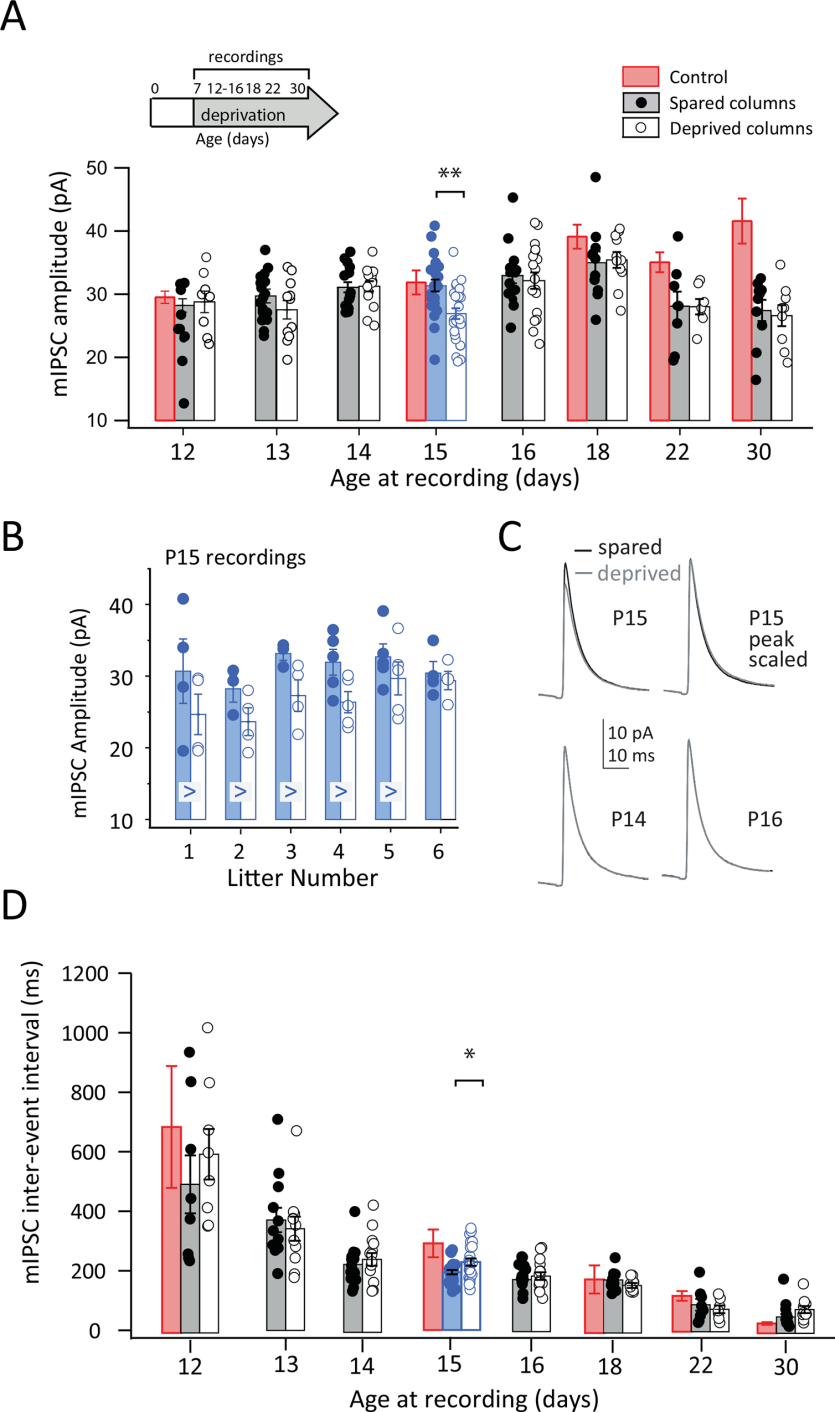
Effect of D-row deprivation from P7 on mIPSCs in deprived vs. spared columns. **A,** mIPSC amplitude for cells in control D columns (no circles) and spared B columns (filled) and deprived D columns (open) from D-row deprived rats. Each circle is one neuron. Bars show mean ± SEM. Blue highlights P15. Means for control, whisker-intact rats (red) are reproduced from Fig 1D. Inset, whisker deprivation and recording ages. **B,** Consistency of deprivation effect at P15 across litters. Each circle is one neuron. >, litter in which mIPSC amplitude was numerically greater in spared vs. deprived columns. **C,** Mean mIPSC waveform across all neurons recorded at P14, P15 and P16. **D,** Mean mIPSC inter-event interval, for cells in control D (no circles), spared B (filled) and deprived D columns (open). Deprived column data in panels A and D were partially shown in Fig 1. Conventions as in (A). * = p < 0.05, ** = p < 0.01

To characterize deprivation effects more sensitively, we measured mIPSCs at more frequent time intervals (every day) and compared them between spared B columns and deprived D columns in the same slices from D-row whisker deprived rats (n = 2–7 rats per age). In spared columns, mIPSC amplitude steadily increased from P12-18, similar to control rats. Beginning at P22, however, mIPSC amplitude decreased in spared columns relative to control columns, with a trend towards decreased amplitude at P22 and a significant reduction at P30 (two-way ANOVA p< 0.001; P22: 35.1 ± 1.6 pA, n = 18 cells; spared: 28.1 ± 2.3 pA, n = 8 cells, 3 rats; P30: control:41.6± 3.7pA, n = 9 cells; spared: 27.4 ± 1.7 pA; n = 10 cells, 7 rats; Bonferroni-corrected t-test, p<0.02) (Fig 2A). Thus, deprivation reduced mIPSC amplitude at P22 and P30 in both deprived and spared columns, relative to control columns in whisker-intact rats.

**Fig 3:**
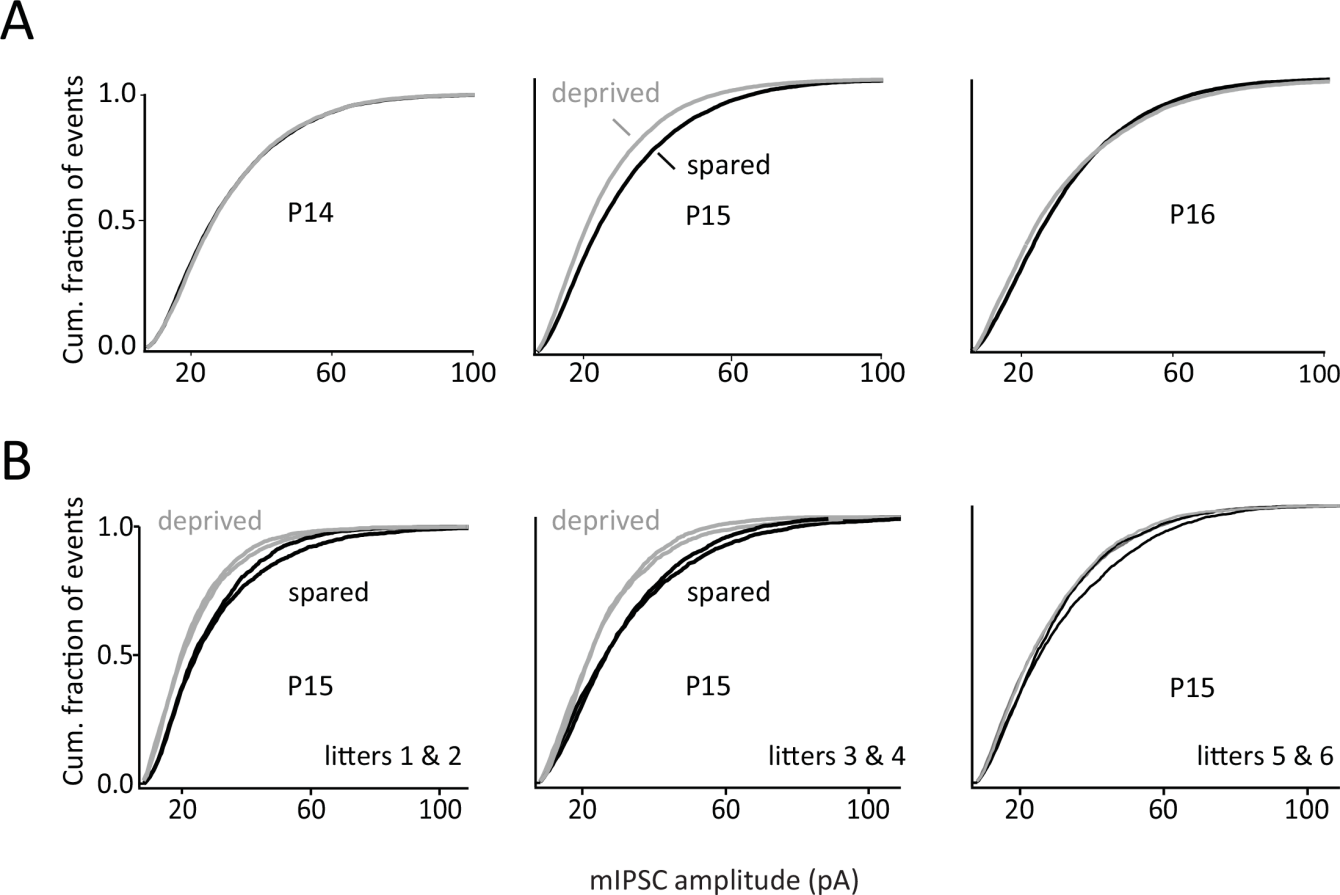
mIPSC amplitude histograms. **A,** Mean cumulative histograms of mIPSC amplitude at P14, P15 and P16, for neurons in deprived D and spared B columns. Each trace is the mean cumulative histogram across all individual neurons. The leftward shift in deprived columns at P15 indicates smaller mIPSCs. **B,** Mean cumulative histograms of mIPSC amplitude compiled separately for each litter. 5/6 litters showed an effect of deprivation on mIPSC amplitude.

In deprived columns, mIPSC amplitude was identical to spared columns at P12-14 (5–7 days of deprivation), but decreased reliably relative to spared columns on P15 (8 days of deprivation) (Fig 2A). This decrease was small (16%) (spared: 31.3 ± 0.9 pA, n = 24 cells; deprived: 26.9 ± 0.9 pA, n = 26 cells, 6 rats), but significant (Bonferroni-corrected t-test, p<0.01). Thus, unlike the P22-30 effect on mISPC amplitude, the reduction at P15 was column-specific, occurring only in deprived D columns, and not in spared B columns. This reduction in mISPC amplitude at P15 occurred in 5 of 6 independent litters tested (Figs 2B and 3B). Surprisingly, this effect was completely confined to P15, with no effect at surrounding ages including P14 and P16 (Figs 2 and 3). This was apparent from mean mIPSC waveforms at P14-16 (Fig 2C) and from the mean cumulative histogram of mIPSC amplitude, which showed a significant leftward shift at P15, but no effect at P14 or P16 (Fig 3A). The effect on mIPSC amplitude at P15 was not associated with any differences in postsynaptic R_input_ (spared: 255.0 ± 14.8 MΩ, deprived: 254.8 ± 15.1 MΩ) or R_series_ (spared: 15.11 ± 0.46 MΩ, deprived: 15.05 ± 0.50 MΩ). Nor did deprivation alter mIPSC rise time (spared: 0.65 ± 0.03 ms, deprived: 0.67 ± 0.03 ms) or decay kinetics (Fig 2C).

Thus, during continuous whisker deprivation, mIPSC amplitude initially increased normally until P14, then exhibited a transient, ~15% reduction in amplitude at P15, which then recovered despite continued deprivation. mIPSC amplitude continued to increase normally until P18. Only at P22 did sustained depression occur. This late depression involved a dynamic reduction in mIPSC amplitude of ~ 25% (Fig 2A).

mIPSC IEI showed the same general developmental decrease in spared and deprived columns as in control rats (Fig 2D). At P15, IEI was slightly greater in deprived vs. spared columns (t-test considering only P15 data, p < 0.05). This effect recovered at P16, and IEI continued to be identical in deprived vs. spared columns through P30. Thus, mIPSC frequency increased normally with development in deprived rats except for a modest transient decrease in frequency at P15. The transient reduction at P15 was also observed in the average inhibitory current per unit time for each cell, computed as mIPSC amplitude * mIPSC frequency (data not shown).

### Deprivation reduces evoked IPSCs selectively at P15

**Fig 4:**
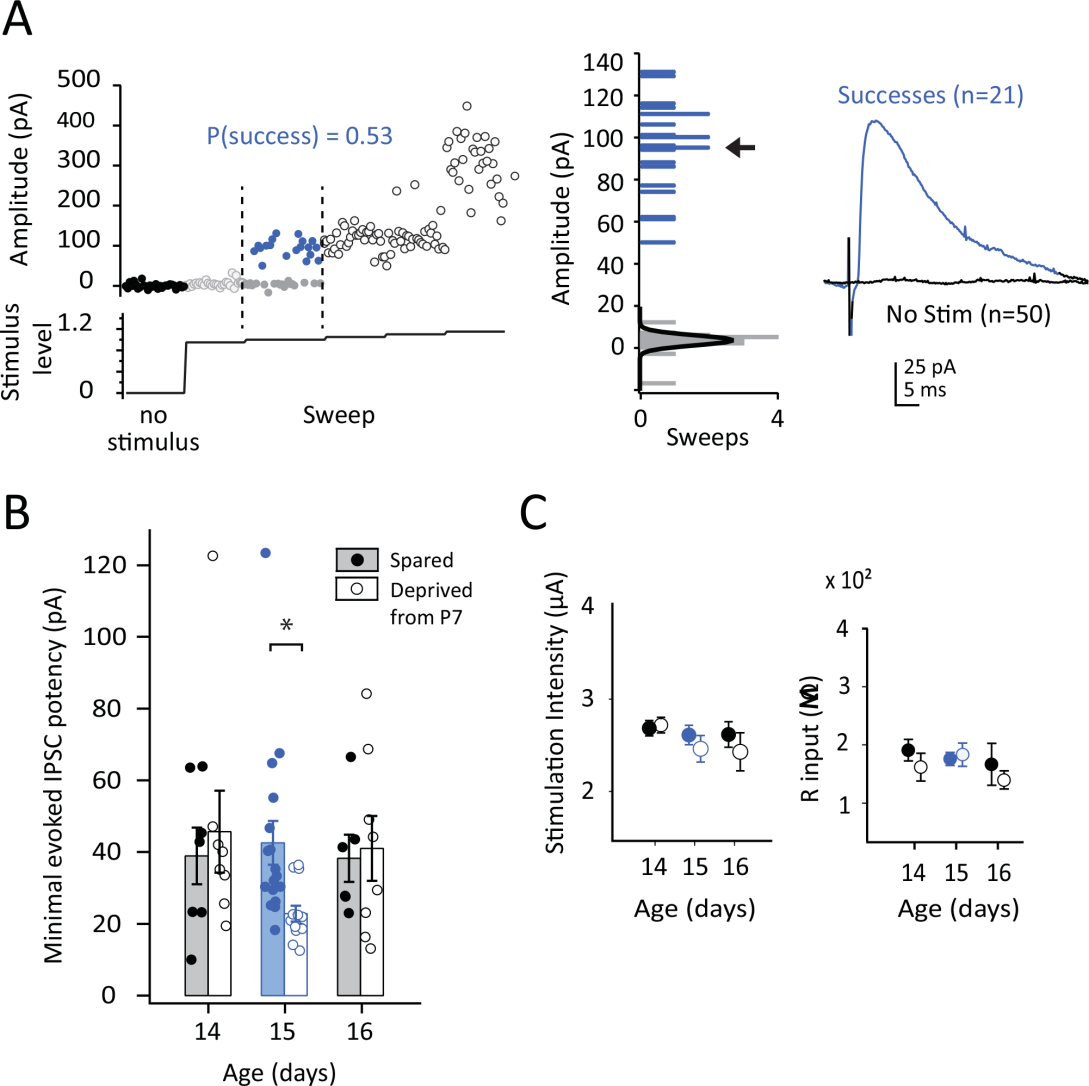
Deprivation effects on evoked IPSC potency at P15. **A,** Example measurement of IPSC potency during minimal stimulation. Stimulus intensity was increased gradually during the experiment (bottom trace). Each circle is IPSC amplitude in one sweep. Black circles, no stimulus. Open gray circles, subthreshold stimulation. Blue and gray solid circles, successes and failures at threshold IPSC stimulus intensity. Open black circles, responses at increasing stimulus intensity. Note that initial increases in intensity above IPSC threshold yielded changes in the probability, but not amplitude, of successes. **Middle,** Amplitudes of successes (blue) and failures (gray) recorded at threshold stimulus intensity for this cell. Arrow, calculated IPSC potency. Black curve shows the no-stimulation noise distribution. **Right,** Mean waveform for successes at threshold stimulus intensity (blue) and for no-stimulation trials (black). **B,** IPSC potency for cells in spared B (filled) and deprived D columns (open). Each circle is one neuron. Bars show mean ± SEM. **C,** Mean stimulation intensity used to measure minimal evoked IPSCs, and mean input resistance, for the IPSC potency experiments. * = p < 0.05

In the rest of the study, we focused on the transient weakening of IPSCs at P15, which has not been previously described. To test whether deprivation also affected evoked inhibitory transmission at P15, we measured IPSC potency during minimal evoked IPSCs using minimal stimulation in L4. We placed a stimulating electrode in L4 in a deprived D or spared B barrel, and evoked GABA-A mediated IPSCs in nearby L4 presumed excitatory neurons using just-suprathreshold stimulation. Monosynaptic fast inhibitory transmission was isolated by including APV (50 μM), NBQX (10 μM), and saclofen (100 μM) in the bath to block fast excitation and GABA-B receptors. Stimulation intensity was adjusted to achieve a failure rate of 40–60%. In this stimulation range, small differences in stimulation intensity yielded changes in the probability, but not amplitude, of successes (e.g., Fig 4A). Because many inhibitory axons make a large number of release sites on target cell somata [31,32], successes and failures are likely to represent stochastic recruitment of spikes in inhibitory axons, rather than stochastic release at individual terminals. The mean amplitude of successes, termed IPSC potency, provides a measure of average inhibitory transmission at one or a few low-threshold inhibitory inputs to a L4 neuron.

Experiments were performed in slices from rats with continuous D-row whisker deprivation from P7. We recorded in spared B and deprived D columns at P14, P15, and P16 (n = 3–5 rats per age). To distinguish successes from failures, we also collected a large number of sweeps without L4 stimulation. This allowed us to measure the noise distribution during no-stimulus trials, and therefore to define successes on stimulation trials as IPSCs that exceeded the no-stimulus distribution in each cell. We calculated the mean amplitude of successes (IPSC potency) for each cell. An example cell is shown in Fig 4A. Deprivation significantly reduced IPSC potency at P15 (spared: 34.1 ± 2.5 pA, deprived: 23.3 ± 1.9 pA, n = 8 cells each, 5 rats, Bonferroni-corrected t-test, p < 0.03), but had no effect on potency at P14 and P16 (Fig 4B). The reduction in IPSC potency at P15 was not due to differences in stimulation intensity or input resistance, which did not vary between B and D columns (Fig 4C). IPSC rise time was also identical in B and D columns at P15 (not shown), suggesting that the reduction in IPSC potency in D columns did not reflect changes in cable properties or differential recruitment of distal vs. proximal (perisomatic) inhibition. When we calculated mean minimal IPSC amplitude, including both successes and failures, a similar trend was found for reduction at P15, but not P14 or P16 (data not shown). Thus, deprivation affected evoked IPSCs, like mIPSCs, transiently at P15.

### Deprivation increases paired pulse ratio (PPR) selectively at P15

As a second test of deprivation effects at P15, we measured short-term plasticity as assessed by paired pulse ratio during sequential IPSCs (PPR; 25 ms interval). Pairs of IPSCs were evoked, with stimulus intensity adjusted to elicit a 40–150 pA for the first IPSC. 3–5 rats were tested per age. Spared B columns showed no paired pulse plasticity, on average, at P12-14 (PPR: 1.21 ± 0.08), and modest paired-pulse depression between P15 and P30 (PPR: 0.83 ± 0.05). This is largely in agreement with previous studies that have observed paired-pulse depression in L4 from P14-P30 [9,13,33].

**Fig 5:**
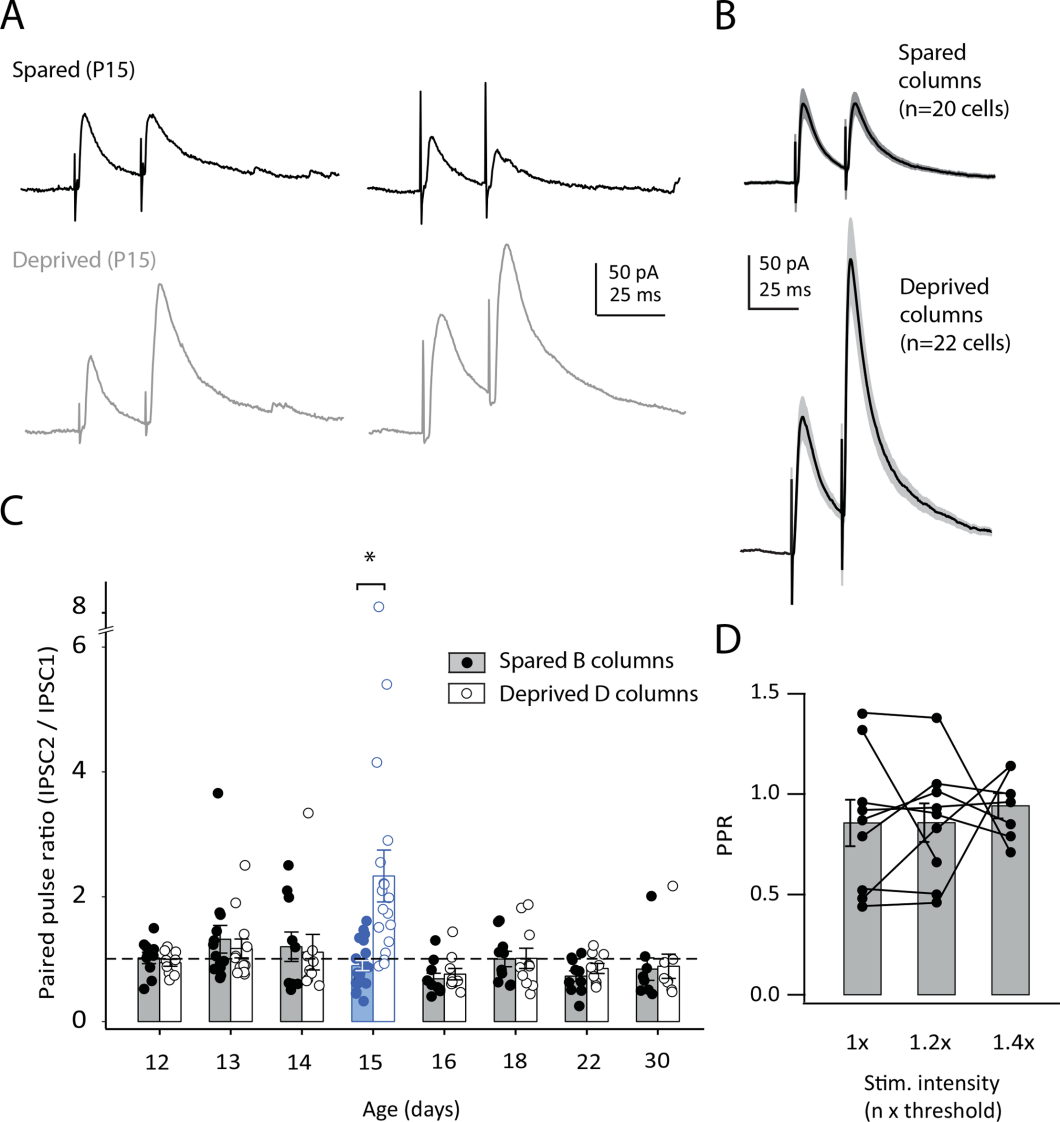
Deprivation effects on PPR of IPSCs. **A,** L4-evoked IPSCs measured at P15 in two example cells in spared B deprived D columns. Holding potential was 0 mV. **B,** Mean IPSC waveform across all neurons at P15 in spared B and deprived D columns. Gray shows ± SEM. **C,** PPR for all cells following D-row deprivation from P7. Each circle is one cell. Bars show mean ± SEM. Blue highlights P15. **D,** PPR as a function of stimulation intensity for 7 cells from control rats. * = p < 0.05

Continuous D-row deprivation from P7 did not alter PPR assessed at P12-14, either in deprived D or spared B columns. However, deprivation caused a pronounced shift to paired pulse facilitation measured at P15, which was selective for deprived D columns. Fig 5A shows two example cells from spared and deprived columns at P15. Fig 5B shows grand average IPSC waveforms for all spared and deprived cells at P15. Quantitative measurement of PPR from IPSC peaks (IPSC2 / IPSC1) showed a selective increase in PPR at P15 in deprived columns (Fig 5C). At P15, mean PPR in deprived columns was 2.3 ± 0.39, compared with 0.88 ± 0.07 in spared columns (n = 22 and 20 cells, 5 rats, respectively; 2-way ANOVA, p<0.001, with Bonferroni-corrected t-test, p<0.05). PPR in deprived columns recovered to normal levels by P16, so that no difference was observed between spared and deprived at P16-30, despite continued deprivation (Fig 5C). To confirm that the shift to paired pulse facilitation at P15 was not due to subtle differences in stimulation intensity, we verified in control animals that PPR was not substantially affected by variations in stimulation intensity (n = 7 cells) (Fig 5D). An increase in PPR is suggestive of a decrease in presynaptic release probability [34]. Thus, both the PPR and evoked IPSC amplitude results indicate that continuous D-row whisker deprivation starting at P7 selectively weakens evoked IPSCs at P15, which then recover despite continued deprivation. P15 is therefore a transient sensitive period for deprivation-induced weakening of both evoked and spontaneous IPSCs.

### Effects of deprivation starting at P12

**Fig 6:**
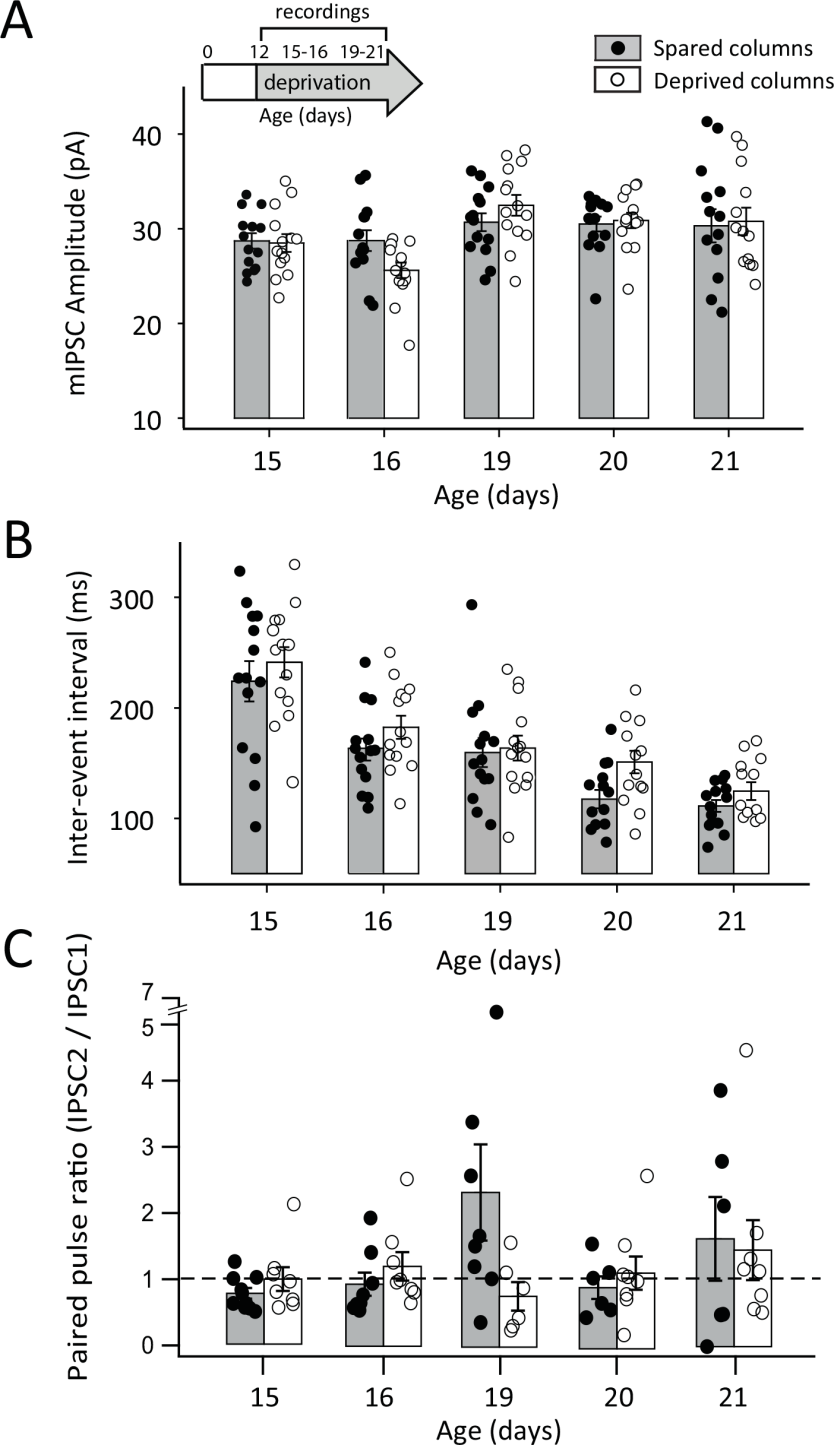
Effects of whisker deprivation starting at P12 on mIPSCs and PPR. **A,** mIPSC amplitude at various ages following continuous deprivation starting at P12, for cells in spared B (filled) and deprived D columns (open). Each circle is one cell. Bars show mean ± SEM. Inset, whisker deprivation and recording ages. **B,** mIPSC inter-event interval. Conventions as in (A). **C,** Mean PPR following continuous deprivation from P12. Conventions as in (A).

Does plasticity of IPSCs at P15 reflect a specific capacity for plasticity at this age, or the expression of plasticity at a specific latency (8 days) after deprivation onset at P7? To address this, we began D-row deprivation at P12, and maintained it continuously until recording. We measured mIPSC amplitude and IEI at P15, 16, 19, 20, and 21 (3 rats from separate litters per age). Deprivation from P12 did not have a significant effect on mIPSC amplitude or IEI at any of the ages tested (Fig 6A and 6B). In separate experiments, we tested whether deprivation from P12 affected PPR of evoked IPSCs, and found no significant difference between spared and deprived columns at any of these ages (2–3 rats from separate litters per age) (Fig 6C). Notably, plasticity was absent both at P15 (the sensitive window identified above for P7 deprivation) and at P20 (8 days after onset of the P12 deprivation). Thus, a modest delay in the onset of deprivation prevented any measurable plasticity in this developmental period, suggesting a defined critical period for these effects.

## Discussion

Sensory experience is important for proper development of inhibitory circuits in S1 [35]. Prior work established that whisker deprivation from P7 to P28 causes a weakening of L4 inhibitory synapses measured at P28 [13], but the dynamics of this process was unknown. Here, we tested whether deprivation causes a gradual accrual of weakening at inhibitory synapses, consistent with a simple arrest of synapse maturation, or whether instead deprivation triggers multiple processes with distinct dynamics, and potentially distinct sensitive periods. We focused on the second and third postnatal weeks, when inhibitory circuits are actively maturing in sensory cortex [5]. Active whisking begins at P12 in rats [22].

We found that continuous whisker deprivation beginning at P7 had no effect on initial development of mIPSC amplitude or kinetics, or on minimal evoked IPSCs or paired pulse ratio for 7 days, through P14. This suggests that early development of average quantal size, release probability, and receptor composition do not require patterned sensory input. Instead, deprivation weakened mIPSCs and evoked IPSCs beginning only at P15, which is 8 days after the onset of deprivation. This weakening was marked by reduced mIPSC amplitude, reduced minimal evoked IPSCs and IPSC potency, and a pronounced shift in paired pulse ratio toward facilitation. Remarkably, these effects rapidly recovered within 24 hrs, despite continued deprivation, so that inhibitory transmission returned to normal by P16, and remained normal until P18 (Figs 2–5). Thus, P15 represents a discrete sensitive period during which sensory loss transiently down-regulates inhibitory transmission in L4, only to subsequently recover. Only at P22 did deprivation drive a sustained weakening of inhibition that persisted to P30, which is the time point at which it has been previously measured in L4 of S1 in mice [13]. We observed only modest effects on mIPSC frequency, with deprivation reducing mIPSC frequency at P15 and P30 but not perturbing the normal developmental increase in mIPSC frequency with age between P12 and P30.

Our findings suggest at least two distinct forms of experience-dependent regulation at developing L4 inhibitory synapses that differ in dynamics, expression mechanisms, and columnar specificity. In a first process, continuous deprivation drives a delayed, transient weakening of IPSCs at P15, which then reverses by P16. In a second process, deprivation beginning at the same age drives an even more delayed weakening of IPSCs at P22, which is sustained at least until P30. In contrast, initial IPSC maturation between P7 and P14 was not affected by deprivation, indicating that whisker experience only begins to affect L4 inhibitory synapses several days after onset of whisking.

### Early IPSC maturation does not require whisker experience

In control rats, we found that mIPSC amplitude and frequency progressively increase from P12 to P30, suggesting increased number and efficacy of GABAergic synapses. This is consistent with previous studies in L4 of S1 [6,36]. Sensory-evoked activity is present in S1 by P7 [37] and whisker deprivation prior to P14 drives plasticity of excitatory circuits and receptive fields in L4 and 2/3 [35,38–40]. Despite this, whisker plucking from P7 did not alter L4 inhibitory synapse development between P7 and P14. This suggests that L4 inhibitory synapses either mature in an activity-independent manner during this period, or that spontaneous activity, residual activity from direct skin contact, or activity evoked by neighboring spared whiskers is sufficient to drive early IPSC maturation. This finding confirms a prior study showing that brief whisker deprivation from P5-P12 does not impair L4 inhibitory synapse function [21]. It is possible that a small fraction of inhibitory synapses may require experience for maturation, but were not detected within the larger synapse population.

It is important to note that we only measured function of inhibitory synapses, not excitatory inputs onto inhibitory interneurons or other aspects of inhibitory network function. Deprivation from P5-12 retards development of excitatory synapses onto L4 inhibitory interneurons [21], and thus is likely to exert profound effects on inhibitory network function, even though inhibitory synapse development proceeds normally.

### Sustained weakening of IPSCs does not occur until P22

Sustained weakening of mIPSCs by whisker deprivation beginning at P7 was not observed until P22, a delay of 15 days (Figs 1 and 2). In deprived animals, mIPSC amplitude was significantly less at P22 and P30 relative to P18, suggesting that deprivation causes an active weakening, rather than arrested development, of inhibitory synapses. This sustained weakening is likely to be the same phenomenon observed at P28 in GAD67-GFP mice [13]. However, the weakening we observed was not specific to deprived columns, was not associated with changes in mIPSC decay kinetics, and was not accompanied by increased PPR, unlike in GAD67-GFP mice [13]. These differences may reflect rat-mouse species differences, or effects of reduced baseline inhibition in GAD67-GFP mice [41]. The finding that sustained weakening did not occur until P22 suggests that brief sensory deprivation during the third week of life would be sufficient to arrest inhibitory synapse development in L4 of S1, as has been observed in L5 of visual cortex [42].

### Transient weakening of IPSCs at P15

We were surprised to observe a delayed, transient weakening of IPSCs at P15, 8 days after onset of deprivation, that spontaneously recovered at P16 despite continued whisker deprivation (Figs 2–5). P15 is substantially later than the classical critical period (P0-P4) for deprivation-induced plasticity of L4 receptive fields [28,39], and lags whisking onset by several days. IPSC plasticity at P15 was characterized by reduced mIPSC amplitude and frequency, reduced IPSC potency in minimal stimulation, and greatly increased paired pulse ratio. These changes were specific to the deprived column, and did not occur in nearby spared columns. This was unlike the late P22 weakening of IPSCs, which occurred in both deprived and spared columns and was not associated with a PPR change. Thus, the P15 and P22 weakening processes appear to be distinct processes with different induction rules and cellular mechanisms.

To test whether the timing of the transient P15 plasticity reflected a special capacity for plasticity at P15 or a requirement for 8 days of deprivation beginning at P7, we shifted the onset of deprivation to P12. This delayed deprivation failed to affect IPSCs measured at either P15 or at P20 (8 days after deprivation onset). Thus, transient weakening was not tied rigidly to 8 days of deprivation. Instead, we hypothesize that P15 is a novel, single-day sensitive period for IPSC plasticity in L4, triggered by deprivation begun early in development. The timing of this sensitive period may be fixed, or may be influenced by external factors in our experiments like progressive reduction in litter size, or potential changes in maternal care related to stress [43].

It is not clear why IPSCs recover at P16, despite continued deprivation. One possibility is that two separate plasticity phenomena are occurring in L4: an early sensitive period at P15 in which deprivation weakens inhibition, followed by a later phase beginning at P16 in which deprivation potentiates inhibitory transmission. This would be similar to inhibitory plasticity reported in L4 of V1 following visual deprivation [44–46].

## Conclusion

Whisker deprivation from P7 does not appear to simply arrest L4 inhibitory synapse development. Instead, our findings indicate at least two distinct plasticity processes: i) a delayed, transient weakening of IPSCs at P15 that is specific to the deprived column and reverses within one day, and ii) sustained IPSC weakening that begins at P22 and is not column-specific. The first, transient weakening process may represent a novel critical period for inhibitory plasticity in L4 of S1. Our findings support the general idea that proper development of inhibitory synaptic transmission in sensory cortex is highly dependent on sensory experience [35,47]. These results suggest that multiple active weakening processes, rather than a general delay in inhibitory synapse development, contribute to deprivation effects on IPSCs in L4 of S1.
